# Prevalence of criminal convictions in Norwegian adult ADHD outpatients and associations with ADHD symptom severity and emotional dysregulation

**DOI:** 10.1186/s12888-021-03223-0

**Published:** 2021-05-03

**Authors:** Espen Anker, Ylva Ginsberg, Trond Heir

**Affiliations:** 1 Oslo ADHD Clinic, Kirkeveien 64B, 0366 Oslo, Norway; 2 Centre for Psychiatry Research, Department of Clinical Neuroscience, Karolinska Institutet & Stockholm Health Care Services, Stockholm, Sweden; 3 University of Oslo, Institute of Clinical Medicine, Oslo, Norway

**Keywords:** ADHD severity, Offending, Deficient emotional self-regulation, Emotional dysregulation, Criminal conviction, Antisocial behaviour, Substance use disorder

## Abstract

**Objectives:**

Attention deficit hyperactivity disorder (ADHD) often co-occurs with antisocial behaviour. Several studies have shown high rates of ADHD among prisoners. However, the prevalence of crime among individuals with ADHD is less known. The aim of the present study was to estimate the prevalence of lifetime criminal conviction (CC) in a clinical sample of adults with ADHD, and the associations with the severity of ADHD and emotional dysregulation (ED).

**Methods:**

Patients were admitted to a private psychiatric outpatient clinic in Oslo between 2014 and 2018. Of the 656 patients diagnosed with ADHD, 629 (95.9%) agreed to participate in the study. CC was determined based on self-reporting of the lifetime history of criminal behaviour. ADHD was diagnosed according to the DSM-5 criteria, and ADHD severity was measured using the Adult ADHD Self-Report Scale (ASRS). ED was assessed by the eight-item version of Barkley’s Current Behaviour Scale - Self-Report (CBS-SR).

**Results:**

The prevalence of self-reported CC in this clinical sample was 11.7% among women and 24.5% among men. CC was associated with hyperactive-impulsive severity (*p* < 0.001) and ED (*p* = 0.006).

**Conclusions:**

The prevalence of self-reported lifetime criminal conviction was high for both genders. CC was associated with symptom severity of hyperactivity-impulsivity and emotional dysregulation. The findings suggest the need for greater research efforts on the avoidance of criminal activity in people with ADHD and targeted intervention for ADHD treatment and CC prevention.

## Introduction

Attention deficit hyperactivity disorder (ADHD) often co-occurs with antisocial behaviour [1, 2]. Several studies have estimated that the prevalence of ADHD among male inmates ranges from 15 to 50% [3–7]. Meta-analyses have indicated an average prevalence of approximately 25% in male inmates [8, 9], and even as high as 40% in female inmates [10]. These rates far exceed the estimated 3–5% prevalence rates of ADHD in the general adult population [11, 12].

Longitudinal studies have found that children with ADHD have a high risk of later antisocial activity and criminal conviction (CC) in adulthood [13, 14]. The long-term outcomes of Danish children with ADHD indicated that nearly half had a history of CC in adulthood [15]. Other studies found girls with ADHD to have seven times higher odds of having an incarceration record than non-ADHD girls, while boys with ADHD had two times higher odds [16]. A Swedish national register-based study found that 15.4% of women and 36.6% of men with ADHD were convicted of any crime [17]. There is a lack of studies comparing CC and no-CC as an outcome for both genders [18].

Knowledge of the prevalence of CC in an ADHD outpatient clinic may be helpful for health authorities to plan appropriate services and help clinicians plan specialized treatments for adult ADHD. Measurements of the prevalence of CC may also contribute to the elucidation of possible causes and the achievement of treatment.

Several studies have indicated that CC and antisocial behaviour are correlated with the severity of ADHD [2, 19–23], and we aimed to confirm this in our study.

Previous studies have found general adjustment problems to be linked to antisocial behaviour in adults with ADHD [24]. Antisocial behaviour is largely mediated by emotional lability and anger problems [25]. Excessive and inappropriate emotional expressions, irritability, and temper outbursts may be recorded as clinical expressions of emotional dysregulation (ED) [26, 27]. ED is a common feature in ADHD, even though it is not part of the criteria of the disorder [28–37]. ED, irrespective of ADHD, is associated with aggressive and violent criminality [38]. ED in adults is clinically similar to oppositional defiant disorder in youth, which has been linked to antisocial behaviour [39, 40]. Whether the severity of ED is associated with CC has yet to be shown.

**The aims** of the present study were to estimate the prevalence of CC in a clinical sample of outpatients with ADHD and to examine its associations with ADHD symptom severity and ED.

## Method

This was an observational cross-sectional clinical study.

### Participants

The study population consisted of adult outpatients aged 18 to 65 who were not imprisoned who fulfilled the diagnostic criteria for a diagnosis of ADHD according to the Diagnostic and Statistical Manual of Mental Disorders, Fifth Edition (DSM-5) [41]. Patients were admitted to a private outpatient non-inmate psychiatric clinic in Oslo, Norway, specializing in the clinical assessment and treatment of adults with ADHD.

Recruitment was conducted between 2014 and 2018. ADHD was assessed using DIVA 2.0 the semi-structured Diagnostic Interview for Adult ADHD, second edition [42]. The assessment was performed by a psychiatrist for all patients included in the study. DIVA 2.0 is a reliable tool for assessing and diagnosing adult ADHD [43]. During the study period, 656 of the assessed patients fulfilled the diagnostic criteria for ADHD and were invited to participate in the study, of whom 65% were self-referred and 35% were referred by healthcare practitioners. None of the participants were using prescribed stimulant medication at the time of assessment. There were no exclusion criteria.

Of the 656 patients (351 men and 305 women) with ADHD, 629 (95.9%) gave written informed consent to participate in the study. The study was approved by the Regional Medical Ethics Committee, South-East Norway, 2015/426. Assessments were carried out in accordance with ethical standards, and the principals of the Declaration of Helsinki.

### Measures

The age of the participants was recorded as their respective numbers of lived years when entering the study. Gender was recorded as ‘woman’ or ‘man’ based on information revealed by the participant. Sociodemographic information was collected as follows: Participant was married or cohabitating. If a participant was living with children inclusive partial custody, but not even though having children somewhere else. Educational level was categorized based on the number of years in education: 12 years or less, 13–15 years, or more than 15 years. Work participation was defined as ‘yes’ if work was reported as the main source of income.

Criminal conviction (CC) was defined as being convicted by the court of any crime under Norwegian law. CC was recorded as positive when participants self-reported CC in response to the following question: ‘Have you ever been in prison or been convicted for any crime?’. We also recorded which crime they were convicted of.

ADHD symptom severity was measured by the Adult ADHD Self-Report Scale (ASRS) Symptom Check List, v1.1 [44, 45] The ASRS is a reliable and valid screening instrument for evaluating ADHD in adults [46]. The 18-item version yields a score ranging from 0 to 72 points. We recorded scores of the subscales of the ASRS questionnaire, i.e., inattentive items (items 1–4 and 7–11) and hyperactivity - impulsivity items (items 5, 6, and 12–18) separately [47].

Emotion dysregulation covers a large variety of emotion dysregulated responses as overwhelming sadness, anxiety, fear, suicidal actions, plural emotional outbursts, and is also linked to several other diagnoses as depression and anxiety (Dvir et al. 2014 [48]), PTSD and personality disorders (Ford & Cortious 2014 [49]), brain injury (Fisher et al. 2015 [50]) or dementia (Ismail et al. 2018 [51]).

Emotion dysregulation in ADHD could alternatively have been assessed by the Impulsivity/Emotional Lability scale from the Conners’ Adult ADHD Rating Scales (CAARS) (Conners et al. 1999 [52]) a 12-item subscale which assesses temper, irritability, stress intolerance and labile mood, but in our paper the definition of emotional dysregulation (ED) was narrowed down to measure explosive anger, temper, or irritability according to Russell Barkley [53]. ED was assessed by using eight relevant items from the larger Current Behaviour Scale - Self Report, known as the Deficient Emotional Self-Regulation (DESR) questionnaire [32, 34, 53, 54]. The eight items were as follows: 1: Quick to get angry or become upset; 2: Easily frustrated; 3: Overreact emotionally; 4: Easily excited by activities going on around me; 5: Lose my temper; 6: Argue with others; 7: Am touchy or easily annoyed by others; and 8: Am angry or resentful. The items were marked by participant as never or rarely (0), sometimes (1), often (2) or very often (3). This yielded a total ED score ranging from 0 to 24.

Alcohol and drug use disorders were diagnosed using a module of the Mini International Neuropsychiatric Interview (M.I.N.I.), Norwegian Translation Version 6.0.0, according to the Diagnostic and Statistical Manual of Mental Disorders, Fourth Edition (DSM-IV) criteria [55, 56]. Dependence and abuse were merged into ‘use’ disorder (as in M.I.N.I. version 7.0/DSM-5), and questions were asked regarding both the last 12-months and lifetime prevalence.

### Statistical analysis

Numbers with percent proportions (%) are reported for all categorical variables. Means with standard deviations (SD) are given for continuous variables. We used chi-square (χ^2^) tests to compare categorical variables and independent sample T-tests to compare continuous variables. We used logistic regression analyses to examine the associations between CC as the outcome variable and age, gender, ADHD symptom severity and ED as the independent variables. To avoid bias in the regression analysis we have decided not to control for probable intermediate variables (or a proxy for an intermediate variable) in the causal pathway between ADHD severity or ED and criminal behaviour (Rothman & Greenland 1998 [57], Gilthorpe et al. 2015 [58], Schisterman et al. 2009 [59]). For example, we have previously published results from the same study sample showing that ADHD severity and ED are associated with alcohol and drug use disorders (Anker et al. 2020 [60]). Both ADHD and ED are early acquired and stable features of a person’s emotional and behaviour reaction patterns less influenced by external circumstances (DSM-5, Eisenberg et al.2010 [61]) and can be interpreted as causal in these relationships. Also, it is well known that alcohol and drug use are strong risk factors for criminal behaviour (Dowden & Brown 2002 [62]). Thus, alcohol and drug use disorders appear to be intermediate variables in the causal pathway between ADHD or ED and criminal behaviour, or they may be a proxy for an intermediate variable in the causal pathway, such as a high-risk social environment. The same argument applies to several variables listed in Table 1, such as education, family relationships and work. Intermediate variables (or a proxy for an intermediate variable), if controlled in an analysis, would usually bias results towards the null, which in the literature is described as a form of overadjustment that should be avoided (Rothman & Greenland 1998 [57], Gilthorpe et al. 2015 [58], Schisterman et al. 2009 [59]).

**Table 1:** Demographics and clinical features in a sample of adult ADHD patients with and without a history of criminal conviction. Participants were recruited in an outpatient psychiatric clinic specialized in examination and treatment of ADHD

	Total sample *n* = 629	Criminal Conviction *n* = 117	No-Criminal Conviction *n* = 512	*p*-value
Age, mean yrs. (SD)	36.7 (11.4)	39.8 (10.0)	35.9 (11.6)	0.020
Gender: Women, n (%)	290 (46.1)	34 (29.1)	256 (50.0)	< 0.001
Men, n (%)	339 (53.9)	83 (70.9)	256 (50.0)	
Years of education: ≤ 12: (%)	321 (51.0)	92 (78.6)	229 (44.7)	< 0.001
13–15: (%)	248 (39.4)	24 (20.5)	224 (43.8)	
> 15: (%)	60 (9.5)	1 (0.9)	59 (11.5)	
Married or cohabitant, n (%)	270 (42.9)	38 (32.5)	232 (45.3)	0.014
Living with children, n (%)	248 (39.4)	30 (25.6)	218 (42.8)	0.003
Work participation, n (%)	370 (58.8)	49 (41.9)	321 (62.7)	< 0.001
Alcohol use disorder lifetime, n (%)	73 (11.6)	29 (24.8)	44 (8.6)	< 0.001
Drug use disorder lifetime, n (%)	165 (26.2)	81 (69.2)	84 (16.4)	< 0.001
Inattentiveness, mean (SD)	27.4 (4.7)	27.9 (5.1)	27.3 (4.6)	0.036
Hyperactivity-Impulsivity, mean (SD)	23.9 (6.6)	26.7 (5.8)	23.3 (6.6)	< 0.001
Emotional Dysregulation, mean (SD)	12.1 (5.6)	14.0 (5.6)	11.8 (5.5)	< 0.001

*p*-values are due to comparison of ADHD patients with and without a history of criminal conviction (chi-square or t-test)

All tests were two-tailed, and differences were considered significant if *p* < 0.05. There were no missing data. All statistical analyses were performed using the software package IBM 2016 SPSS version 22 [63]. We used Cronbach’s alpha statistic to assess the internal consistency reliability of the eight items from the DESR scale. Cronbach’s alpha for the eight scale items in our sample was 0.86 indicating high internal consistency.

## Results

Table 1 shows participants with and without a history of criminal conviction (CC). Those with a history of CC were older, were more often men, had a lower educational level, were less involved in family life and work, and had a higher risk of a history of alcohol or drug use disorders than those without a history of CC.

The prevalence of CC was 11.7% (*n* = 34) in women and 24.5% (*n* = 83) in men.

Among women (*n* = 290), the most common conviction was for selling or possessing drugs (*n* = 18; 6.2%), followed by violence, (*n* = 6; 2.1%), traffic crime (n = 6; 2.1%), theft (*n* = 2; 0.7%), and mixed or multiple convictions (n = 2; 0.7%).

Among men (*n* = 339), the most common conviction was for selling or possessing drugs (*n* = 32; 9.4%), followed by violence, (*n* = 24; 7.1%), traffic crime (*n* = 14; 4.1%), theft (*n* = 5; 1.5%), and mixed or multiple convictions (*n* = 8; 2.4%).

Hyperactive-impulsive symptom severity and ED were higher in patients who had a history of CC (Table 1).

Table 2 shows associations between criminal conviction (CC) as the outcome variable and age, gender, ADHD symptom severity and emotional dysregulation (ED) as independent variables. Higher levels of hyperactivity-impulsivity symptoms and ED were significantly associated with CC bivariate as well as in multivariate logistic regression model adjusted for age and gender.

**Table 2:** Associations between criminal conviction (CC) as the outcome variable and age, gender, ADHD symptom severity and emotional dysregulation (ED) as independent variables in a clinical sample of adult females (n = 290) and males (n = 339) with ADHD

	Unadjusted			Adjusted		
	OR	95%CI	p-value	OR	95%CI	p-value
Age (increase in 10 years)	1.34	1.13–1.60	0.001	1.32	1.10–1.59	0.003
Gender (Men v. Women)	2.44	1.58–.77	< 0.001	3.38	2.12–5.39	< 0.001
ADHD symptom severity						
Inattentiveness	1.03	0.98–1.07	0.28	0.96	0.91–1.02	0.17
Hyperactivity-Impulsivity	1.09	1.05–1.12	< 0.001	1.09	1.04–1.13	< 0.001
Emotional dysregulation	1.08	1.04–1.12	< 0.001	1.07	1.02–1.12	0.006

Results are given as odds ratios (ORs) with 95% confidence intervals (95%CI) in bivariate (unadjusted) and multivariate (adjusted) logistic regression models

There were no significant interactions among age, gender, hyperactive-impulsive symptom severity and ED.

## Discussion

The study revealed a high incidence of crime among adult ADHD patients. The 11.7 and 24.5% life-time prevalence rates of criminal conviction (CC) in female and male ADHD patients respectively, were considerable higher than the correspondingly 1 and 5% rates for women and men in the general Norwegian population [64]. High risk of crime in people with ADHD is also known from other Scandinavian countries with relatively low incidences of crime in the general population [17, 65].

The two or threefold higher risk of CC among men versus women is in line with other studies of ADHD patients [7, 16, 65]. The ratio is similar to that in the general population [64, 66], which indicates that gender differences are not affected by ADHD.

The finding that CC was associated with the severity of hyperactive-impulsive symptoms is in line with several studies that have shown associations between the severity of ADHD symptoms and different aspects of antisocial behaviour [25, 67–71]. This finding is also consistent with longitudinal studies of children with ADHD showing that the severity of hyperactivity-impulsivity symptoms was associated with later occurrence of CC [64, 72]. It is likely that restlessness and impulsivity symptoms may result in less well-considered behaviour that may also include violation of the law. It is also possible that criminal acts by people with hyperactive-impulsive symptoms are more due to sensation and novelty seeking [73, 74] and less planned and proactive and that people with ADHD are therefore more likely to be convicted [75].

With this aspect in mind, it seems meaningful to address the severity of ADHD as emphasized in the DSM-5 [41] as opposed to categorizing ADHD by sub-type as suggested in the DSM-IV [76]. The strong association between CC and hyperactive-impulsive severity also underlines the importance of treating ADHD as a dimensional diagnosis and expecting more antisocial behaviour in patients presenting with high levels of hyperactive-impulsive symptoms.

The concept of emotional dysregulation (ED) has different aspects, with both bottom-up strong emotional responses and top-down poorer regulation of emotions. The DESR questionnaire is mostly focused on bottom-up emotionality, which has been understood as ED. In our sample, CC was associated with the severity of ED, independent of hyperactive-impulsive severity. We suggest that knowledge on ED does add significantly to the understanding of CC in people with ADHD, First, ED is a common feature of ADHD, even though it is not part of the diagnostic criteria [28–37, 77]. Second, ED contributes significantly to general impairments in patients with ADHD [78] and has an independent effect on general social problems associated with ADHD [34, 79]. Third, aggression in childhood has been found to predict criminality later in life [72], and personality traits of negative emotionality have been linked to criminal activity [80]. Adding ED severity to the concept of ADHD is in line with Reimherr et al., who emphasize a two-factor solution for ADHD subgroups, with a presentation consisting solely of inattention and a more comprehensive hyperactivity-impulsivity-emotional dysregulation presentation [81].

We found a strong relationship between all background variables and CC. The association between low education and CC is notable since people with ADHD have lower levels of education than the general population [82–84]. Additionally, drug use disorder was correlated with CC, which is worth noting since ADHD patients have higher incidences of drug use than the general population [25, 60, 65, 85, 86].

The relationship between crime and substance use disorders may be due to several mechanisms. It is reasonable that substance use and addiction can lead to crime. On the other hand, criminal activity can provide closer contact with intoxicants and drug environments. In addition, there may be common underlying factors that increase the risk of both substance use and crime. The fact that we exclusively had lifetime prevalence for both substance use and CC, and lacked hypotheses of cause-effect, led us not to adjust for substance use in the regression model.

Early identification of ADHD and disruptive behaviour, with subsequent multimodal interventions might reduce the risk of a criminal trajectory. Supporting people with ADHD in attaining higher education and work and avoiding drugs is probably also a good approach to prevent CC.

There are some methodological limitations to consider in this study. CC was measured based on self-report, which may have resulted in under-reporting. Patients attending a private clinic that is not governmentally funded may not be representative of patients with ADHD in general. They may have a higher socioeconomic status and be less impaired than patients in public outpatient clinics or prison inmates. Additionally, the prevalence rates of criminality and morbidity in this population may not be representative of those of the total ADHD patient population. We assume that differences in sample selection may primarily affect the prevalence rates and, to a lesser extent, their associations [87, 88]. The associations with CC should therefore be more generalizable.

The cross-sectional observational design limited the interpretation of causal relationships. We assume ADHD and ED are traits that develop in childhood before the criminal minimum age, which is 15 years in Norway, which may therefore indicate the direction of causality.

## Conclusion

In this clinical sample the prevalence of criminal conviction (CC) was high for both genders compared to the prevalence rates reported in the general population. CC was associated with the severity of hyperactivity-impulsivity, as well as emotional dysregulation, which indicates that knowledge on both features contributes to the understanding of CC in ADHD.

### Clinical implication

Criminal behaviour is common in adults with ADHD. The findings call for research efforts to prevent criminal activity in people with ADHD. The association of higher severity of hyperactivity-impulsivity symptoms and emotional dysregulation with CC suggests that clinical manifestations should be treated carefully. Early identification as well as biological- and psychological approaches in the prevention and treatment of ADHD and externalizing behaviour should be performed early in childhood and followed up in adulthood by professionals with specific knowledge of treatment and behavioural interactions with the environment. Furthermore, targeted interventions should focus on the preventive effect of academic achievement and higher education for adolescents with ADHD, which could be crucial in avoiding illegal drug use, antisocial behaviour, and criminal activity.

## Data Availability

Data were collected from a private psychiatric outpatient ward in Oslo. Public availability of the data would compromise privacy of the respondents. According to approval from the Norwegian Regional Committee for Medical and Health Research Ethics, the data must be stored properly in line with the Norwegian Law of Privacy Protection. However, anonymized data are freely available to interested researchers upon request, pending ethical approval from the ethics committee. Interested researchers can contact project leader Espen Anker (espen.anker@online.no) with requests for the data.

## Abbreviations

ADHD: Attention Deficit Hyperactivity Disorder
ED: Emotional Dysregulation
MINI: Mini International Neuropsychiatric Interview
ASRS: Adult ADHD Self-Report Scale.
CC: Criminal Conviction

## Declarations

We confirm that all methods were performed in accordance with relevant guidelines and regulations.
